# Differences in the intraspecies copy number variation of *Arabidopsis thaliana* conserved and nonconserved miRNA genes

**DOI:** 10.1007/s10142-023-01043-x

**Published:** 2023-04-10

**Authors:** Anna Samelak-Czajka, Pawel Wojciechowski, Malgorzata Marszalek-Zenczak, Marek Figlerowicz, Agnieszka Zmienko

**Affiliations:** 1 Institute of Bioorganic Chemistry, Polish Academy of Sciences, 61-704 Poznan, Poland; 2 Institute of Computing Science, Faculty of Computing and Telecommunications, Poznan University of Technology, 60-965 Poznan, Poland

**Keywords:** MicroRNA, miRNA, Copy number variation, Expression, Transposons, ATHILA

## Abstract

MicroRNAs (miRNAs) regulate gene expression by RNA interference mechanism. In plants, miRNA genes (*MIR*s) which are grouped into conserved families, i.e. they are present among the different plant taxa, are involved in the regulation of many developmental and physiological processes. The roles of the nonconserved *MIR*s—which are *MIR*s restricted to one plant family, genus, or even species—are less recognized; however, many of them participate in the responses to biotic and abiotic stresses. Both over- and underproduction of miRNAs may influence various biological processes. Consequently, maintaining intracellular miRNA homeostasis seems to be crucial for the organism. Deletions and duplications in the genomic sequence may alter gene dosage and/or activity. We evaluated the extent of copy number variations (CNVs) among *Arabidopsis thaliana* (Arabidopsis) *MIR*s in over 1000 natural accessions, using population-based analysis of the short-read sequencing data. We showed that the conserved *MIR*s were unlikely to display CNVs and their deletions were extremely rare, whereas nonconserved *MIR*s presented moderate variation. Transposon-derived *MIR*s displayed exceptionally high diversity. Conversely, *MIR*s involved in the epigenetic control of transposons reactivated during development were mostly invariable. *MIR* overlap with the protein-coding genes also limited their variability. At the expression level, a higher rate of nonvariable, nonconserved miRNAs was detectable in Col-0 leaves, inflorescence, and siliques compared to nonconserved variable miRNAs, although the expression of both groups was much lower than that of the conserved *MIR*s. Our data indicate that CNV rate of Arabidopsis *MIR*s is related with their age, function, and genomic localization.

**Supplementary Information:**

The online version contains supplementary material available at 10.1007/s10142-023-01043-x.

## Introduction

Since the discovery of the first microRNAs (miRNAs) in *Caenorhabditis elegans* three decades ago (Lee et al. 1993; Wightman et al. 1993), the knowledge of the biology of these small, single-stranded RNA molecules has grown significantly. The interest in miRNAs increased with the discovery that the mechanism of regulating gene expression by RNA interference may be ubiquitous among other organisms, rather than exclusive to *C. elegans*. In fact, up to date, miRNAs have been shown to be widespread among eukaryotes. In 2022, the miRBase database (Release 22.1), which is the primary online catalogue of miRNA sequences and annotations, contained 38,589 hairpin precursors from 271 organisms, including 82 plant species (Kozomara et al. 2019).

MiRNA genes (*MIRs*) are transcribed by RNA polymerase II to pri-miRNA precursors, which possess a characteristic intramolecular hairpin structure. Plant pri-miRNAs are subsequently processed by RNase III nuclease, named Dicer-like protein (DCL), in a two-step cleavage process (Kurihara and Watanabe 2004). During the first cut, the hairpin structure is cut off from the transcript. Next, the resulting precursor miRNA hairpin (pre-miRNA) is cut again to form a 21-22-nucleotide-long miRNA/miRNA* duplex, which is subsequently methylated on its 3’-termini by HEN1 methyltransferase (Yu et al. 2005). The 3'-methylated duplex is exported to the cytoplasm, where the miRNA strand is loaded onto the RNA-induced silencing complex (RISC) and guides gene silencing, while the other strand is ultimately degraded. Most plant miRNAs are 21-nucleotide-long molecules (Budak and Akpinar 2015; Yu et al. 2017; Song et al. 2019). *MIRs*, which produce similar or identical mature miRNAs, are grouped into gene families. Some of these families are conserved among the different taxa within the plant kingdom, including distantly related species. Other, nonconserved *MIR*s families are restricted to a narrow phylogenetic taxon, e.g. family, genus, or even species (Qin et al. 2014). There are also singletons, which are unique *MIRs*, present in specific plants.

**Fig. 1:**
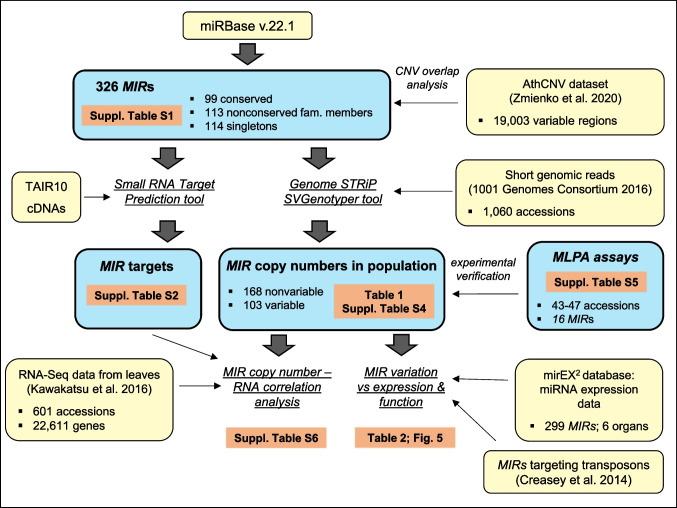
Schematic depiction of data sources and *MIR* analysis conducted in this study

Maintaining genetic variability is crucial for sustaining the adaptive potential of the species and its ability to respond to changing conditions, inhabit new environments, and consequently shift or expand the species range (Mitchell-Olds and Schmitt 2006; Barrett and Schluter 2008). Among the factors, which maintain genome plasticity, are copy number variations (CNVs), i.e. large deletions and duplications of DNA segments. CNVs may directly affect the gene dosage and thus exert phenotypic changes. A growing line of evidence indicates that some *MIRs* present intraspecies copy number diversity, and this diversity may be associated with the phenotypic variation. Marcinkowska et al. (2011) identified 221 CNV-miRNAs in the human genome, 38 of which were located in chromosomal regions implicated in microdeletion/microduplication syndromes. Additionally, *MIRs* copy number alterations were revealed in a range of human diseases, including cancer, autism, schizophrenia, or intellectual disabilities (Vaishnavi et al. 2013; Qiao et al. 2013; Persengiev et al. 2013; Warnica et al. 2015; Anauate et al. 2019; Vischioni et al. 2022). However, to our knowledge, the extent of *MIRs*’ copy number polymorphism in the model dicot *Arabidopsis thaliana* (hereafter named Arabidopsis) and their potential impact on gene expression variation has not been investigated so far. To fill this gap, we evaluated whether Arabidopsis *MIR* conservation and genomic distribution are linked to their copy number variation. We analysed the overlap between *MIRs* and CNV regions in the Arabidopsis genome, assessed *MIR* copy number diversity in ~ 1,000 accessions, by the short-read sequencing data-based analysis, and evaluated the association of *MIR* variability with miRNA expression profiles in the reference accession, Col-0. The schematic representation of our bioinformatic and experimental analyses is presented in Fig. 1. We demonstrate that the nonconserved *MIRs*, which are thought to be involved mainly in plant adaptation and stress-related responses, display higher intraspecies variation than the conserved *MIRs*, which frequently regulate the essential developmental traits. We also discuss the potential contribution of transposable elements (TEs) to intraspecies variation of Arabidopsis *MIR*s.

## Results

### Arabidopsis conserved and nonconserved *MIR*s differed in their localization and genomic context

**Fig. 2:**
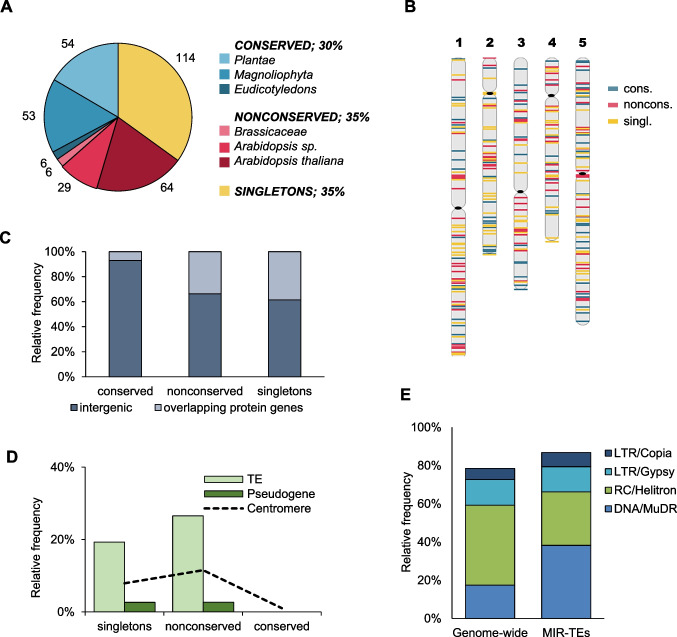
*MIR*s in the Arabidopsis genome. **A** Conservation level of *MIR*s found in Arabidopsis. **B** Distribution of *MIR*s along the chromosomes. **C** Relationship between *MIR* conservation and their overlap with the protein-coding genes. **D** Frequency of *MIR*s localized in the centromeres and/or overlapping pseudogenes and TEs. **E** Frequency of TEs from various TE families in Arabidopsis genome (genome-wide) and among the TEs which overlap with *MIR*s (*MIR*-TEs). For clarity, only four most abundant *MIR*-TE families are presented

According to the miRBase database v. 22.1, there were 326 known miRNA stem-loop precursors of 428 mature miRNAs in Arabidopsis (Kozomara et al. 2019). Of them, 99 (30%) belonged to one of 29 conserved families, 113 (35%) were members of 72 nonconserved families (restricted to Brassicaceae), and 114 (35%) were singletons, individual *MIRs*, with unique mature miRNA sequence, which so far have been found only in Arabidopsis (Fig. 2A; Additional file 1: Supplementary Table S1). It should be noted that this classification is subject to change in the future, as additional *MIR*s may be discovered in various plant genomes. We next used the annotation of the Col-0 accession reference genome (TAIR10 assembly) to evaluate *MIRs* distribution and their overlap with other genetic elements. We also performed bioinformatic predictions of *MIR* targets in Arabidopsis cDNA library, using small RNA target prediction tool (Jones-Rhoades and Bartel 2004; Nakano et al. 2020). This search resulted in predicting from 1 to 2828 potential targets for 311 *MIR*s (median number of targets was 6) (Additional file 1: Supplementary Table S2). The *MIRs* were uniformly distributed along all five chromosomes (Fig. 2B), and the majority of them were fully intergenic, i.e. localized outside of the protein-coding genes. However, the rate of conserved intergenic *MIRs*, 93% (92), was significantly higher, compared to intergenic members of the nonconserved *MIR* families, 66% (75), or singletons, 61% (70) (Fig. 2C). Eighty-nine *MIRs* overlapped with the protein-coding gene loci by at least one nucleotide. Among them, only five *MIRs* from the nonconserved families and 15 singletons partially or fully overlapped with the coding sequence, while the remaining ones (7 conserved, 33 from nonconserved families, and 29 singletons) were located in the noncoding regions of the gene models.

Twenty-three *MIRs* (1 conserved, 13 from nonconserved families, and 9 singletons) were located in the centromeres (Fig. 2D). Eight centromeric *MIR*s were derived from *ATHILA* retrotransposons (members of the LTR/Gypsy superfamily) which are well-known for their association with the centromeres and heterochromatin (Pereira 2004; Slotkin 2010). Recently, a new genomic assembly of Col-0 accession was obtained with the use of long-read sequencing (Naish et al. 2021). In this assembly, named Col-CEN, all centromeres were fully resolved, and 53 intact as well as 20 fragmented centromeric *ATHILA* retrotransposons were annotated. By LTR comparisons, the authors showed that the centromeric *ATHILA* were young, with, on average, 98.7% LTR sequence identity. Moreover, postintegration duplication events of *ATHILA5* and *ATHILA6A* in centromere 5 were detected. Members of MIR854 family were annotated within *ATHILA6A* elements. Since much of the centromeric sequence has been missing from the current reference genome (Naish et al. 2021), we hypothesized that additional yet unannotated MIR854 loci may be present in Col-CEN assembly. To check for this possibility, we performed blast searches of TAIR10 and Col-CEN assemblies, using *ath-MIR854b* precursor sequence as a query, and we recovered all hits with 100% query coverage and at least 97% identity. For TAIR10, this resulted in exactly 5 hits, corresponding to the known MIR854 family members. In Col-CEN, however, 16 hits were recovered, mostly in centromere 5. All but one of these potential MIR854 precursors were positioned inside the intact centromeric *ATHILA6A* retrotransposons (Additional file 1: Supplementary Table S3). This finding confirmed that *ATHILA6A* invasion and subsequent duplication directly contributed to the expansion of MIR854 family in Arabidopsis.

Genome-wide, 52 *MIR*s were fully or partially overlapped by one or more TEs. Significantly, none of them belonged to conserved *MIR*s, compared to 22 singletons (19%) and 30 *MIRs* from 8 nonconserved families (27%) (Fig. 2D). Four TE superfamilies with the highest number of overlapping *MIR*s included DNA/MuDR elements (26 TEs overlapping 17 *MIRs*), RC/Helitrons (19 TEs overlapping 17 *MIR*s), LTR/Gypsy elements (9 TEs overlapping 8 *MIR*s), and LTR/Copia elements (5 TEs overlapping 3 *MIR*s). Therefore, both class I and class II TEs could serve as sources of new *MIR*s. However, TEs belonging to DNA/MuDR family were 2.2 times more frequent among the *MIR*-overlapping TEs, compared to their genome-wide occurrence (Fig. 2E). Among them, *ATMU3N1* and *ATMU10*, two types of the miniature inverted-repeat transposable elements (MITEs), were especially abundant and overlapped with the members of *MIR5635* and *MIR5645* families, respectively. One of the acknowledged models of de novo miRNA biogenesis assumes that MITEs, which are truncated derivatives of autonomous DNA transposons and contain terminal inverted repeats, could produce an imperfect hairpin when transcribed (Piriyapongsa and Jordan 2007). Accordingly, a recent study showed that MITEs constituted the dominant source of de novo miRNAs in angiosperms, including Arabidopsis (Guo et al. 2022), which could explain why we observed the enrichment in DNA/MuDR elements among the *MIR*-overlapping TEs. On the contrary, RC/Helitrons, which constitute the most abundant TE superfamily in Arabidopsis, both in terms of TE copy number and the total genome coverage (Quesneville 2020), were 1.5 times less frequent among the *MIR*-overlapping TEs. Helitrons are DNA transposons, which transpose through a putative rolling circle mechanism and do not contain terminal inverted repeats. According to the plant transposable element-related miRNA database, only one Helitron-associated *MIR* was found among the nine non-Brassicaceae genomes (Lorenzetti et al., 2016). Similarly, in the human genome, one RC/Helitron-derived *MIR* was identified in a computational search (Gim et al. 2014). On the other hand, *Brassica rapa* genome showed highest Helitron density among 44 plant species surveyed in another study, and numerous Helitron-derived *MIR*s were predicted in this plant (Fu et al. 2013; Hu et al. 2019).

### *MIR*s associated with protein-coding genes displayed little intraspecies copy number variation

**Fig. 3:**
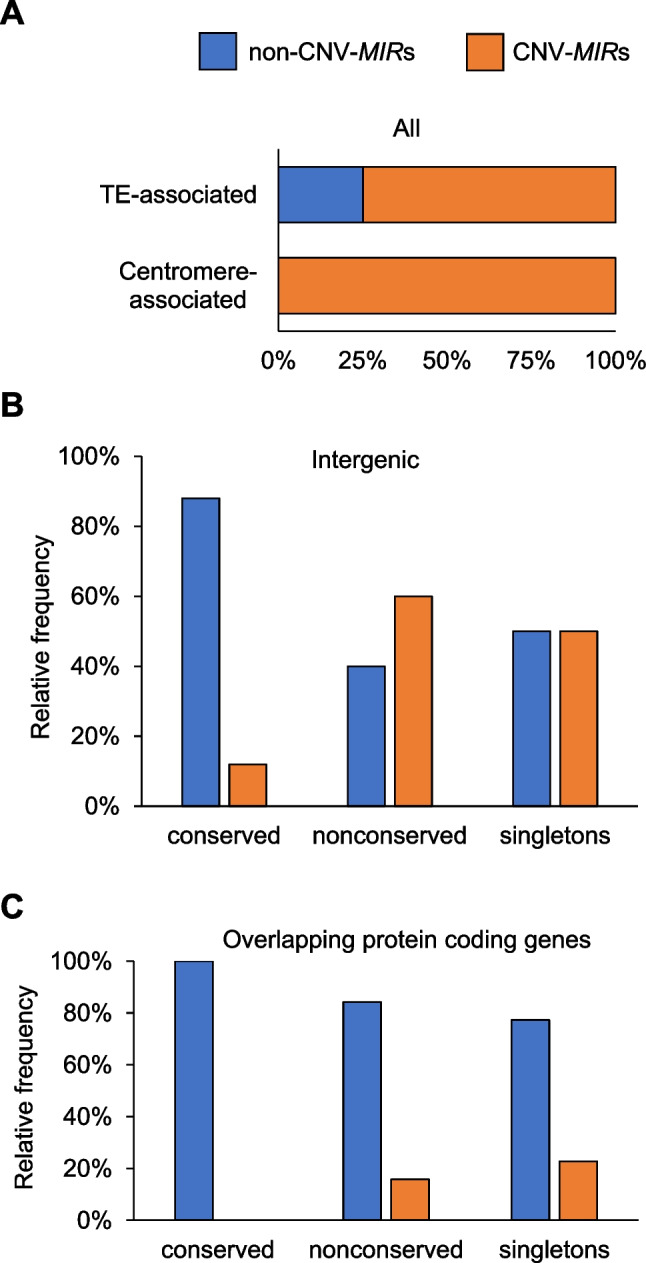
Nonuniform overlap of *MIR*s by CNV segments in Arabidopsis genome. **A** Fractions of CNV-*MIR*s associated with TEs and the centromeres. **B** Fractions of conserved and nonconserved CNV-*MIR*s associated with intergenic regions. **C** Fractions of conserved and nonconserved CNV-*MIR*s associated with the protein-coding genes

To evaluate the rates of intraspecies variability of Arabidopsis *MIR*s, we first compared the genomic coordinates of all *MIRs* and the common Arabidopsis CNVs, retrieved from the AthCNV catalogue (Zmienko et al. 2020). Nearly one-third of *MIRs* (107) overlapped with or were fully contained within the variable regions (CNV-*MIRs*), including 75% of TE-associated *MIRs*, as well as all *MIRs* located in the centromeres (Fig. 3A). Altogether, CNV-*MIRs* included 40% of all singletons, 45% of all *MIRs* from the nonconserved families, and only 11% of the conserved *MIRs*. The differences in CNV overlap between conserved and nonconserved *MIR*s were most highlighted in the intergenic regions: 60% of genes from the nonconserved families and 50% of singletons were localized within CNVs, compared to only 12% of the conserved *MIRs* (Fig. 3B). However, among the *MIRs* overlapping protein-coding genes, CNV-*MIRs* constituted only 16% of the nonconserved families’ members, 23% of singletons, and none of the conserved *MIRs* (Fig. 3C). Based on these observations, we concluded that the conserved *MIRs* were preferentially associated with the nonvariable regions, while there was no such bias for the nonconserved *MIR* families and singletons. Moreover, overlap with the protein-coding gene was the factor limiting the variability of all *MIRs*.

However, the fact that a *MIR* was overlapped by a CNV region did not indicate which accessions carried duplications or deletions and whether *MIR* variation among the accessions was high or low. Therefore, to get a higher-resolution view of the *MIR*s variability, we performed read depth analysis and evaluated the copy numbers of individual *MIR*s in each of 1060 natural accessions from the 1001 Genomes collection (Handsaker et al. 2015; 1001 Genomes Consortium 2016; Zmienko et al. 2020). For this purpose, each region subjected to genotyping was centred on a single *MIR* locus, and its size was adjusted to the *MIR* length (usually equal to the length of the stem-loop precursor deposited in the miRBase), additionally extended by 300-bp flanks from both sides. The purpose of adding the flanks was to obtain a better coverage by short-read sequencing data. However, it is worth noting that, according to recent estimations, many Arabidopsis pri-miRNAs are much longer than reported in the miRbase, and the average length of pri-miRNAs exceeds 1.2 kb in this plant (Lauressergues et al. 2022). Therefore, extending the genomic regions beyond the formal coordinates of annotated *MIR* loci additionally allowed us to cover their expected true size more accurately. For the analysis, *MIR*s which overlapped each other (there were seven such pairs) were grouped, which resulted in a total of 319 regions subjected to genotyping. For 48 of them, the analysis failed, or they were filtered out due to abnormal copy number distributions (see Materials and Methods). The set of filtered out genes included 18 out of a total of 20 *MIR*s located within the so-called hotspots of rearrangements—highly variable regions in the Arabidopsis genome, the presence of which was revealed only recently by PacBio long-read sequencing and de novo assembling the genomic data (Jiao and Schneeberger 2020). We note that half of the TE-associated *MIR*s failed to be genotyped at all or were removed following our filtration procedure. This included, e.g., all members of the centromeric MIR854 family and reflects known difficulties in unique mapping of short reads to the repetitive elements in the genome, as well as shortcomings of the current reference genome (Chu et al. 2021, Naish et al. 2021).

For 271 successfully genotyped *MIR*s (Additional file S4: Supplementary Table S4), we applied highly restrictive thresholds that would allow us to detect copy number losses and gains (copy number estimates lower than 0.3 or at least 4.0, respectively). According to these criteria, 103 *MIR*s (23 conserved, 35 from nonconserved families, and 45 singletons) displayed copy number changes—we further referred to them as variable *MIR*s (Table 1). The remaining 168 *MIRs* (72 conserved, 45 from nonconserved families, and 51 singletons) showed no changes in the copy number in the investigated accessions—we referred to them as nonvariable *MIR*s. Consistent with the AthCNV overlap analysis, 153 of the nonvariable *MIR*s were localized outside the common CNVs. *MIR*s associated with protein-coding genes showed very little variation in the analysed population—56 out of 75 genotyped *MIR*s overlapping with protein-coding genes were nonvariable (75%). Protein-coding genes are known to be depleted in CNV regions (Zmienko et al. 2020); therefore, we believe that their overlap with *MIR*s was the main factor affecting *MIR* variability. Unfortunately, the molecular function or biological process Gene Ontology classifications are unavailable for two-thirds of protein-coding genes overlapping with *MIR* loci; therefore, it is difficult to assess whether their presence acted against *MIR* variation (Thomas et al. 2022). We noted that most *MIR*s predicted to directly target the overlapping genes were nonvariable (Additional file 1: Supplementary Table S4); however, they are usually predicted to have multiple targets (Additional file 1: Supplementary Table S2), and few of them have been validated. Whether the low variability of individual *MIR*s might be related to their function in posttranscriptional gene silencing remains to be established.

**Table 1:** Copy number nonvariable and variable *MIR*s grouped by family

Conserved			Nonconserved					
Family	Nonvariable	Variable	Family	Nonvariable	Variable	Family	Nonvariable	Variable
MIR156	7	3	MIR5645	0	4	MIR400	1	0
MIR166	6	3	MIR5020	0	3	MIR402	1	0
MIR169_2	3	3	MIR447	1	2	MIR4228	1	0
MIR169_1	5	2	MIR5635	1	2	MIR4240	1	0
MIR172	3	2	MIR3932	0	2	MIR4245	1	0
MIR397	0	2	MIR405	0	2	MIR5595	1	0
MIR395	5	1	MIR5638	0	2	MIR771	1	0
MIR399	5	1	MIR773	0	2	MIR822	1	0
MIR160	2	1	MIR841	0	2	MIR823	1	0
MIR398	2	1	MIR158	1	1	MIR824	1	0
MIR168	1	1	MIR2112	0	1	MIR825	1	0
MIR2111	1	1	MIR4221	0	1	MIR829	1	0
MIR390	1	1	MIR4227	0	1	MIR831	1	0
MIR167_2	0	1	MIR4243	0	1	MIR834	1	0
MIR159	6	0	MIR5649	0	1	MIR835	1	0
MIR171_1	4	0	MIR5998	0	1	MIR837	1	0
MIR164	3	0	MIR781	0	1	MIR838	1	0
MIR167_1	3	0	MIR8167	0	1	MIR839	1	0
MIR393	2	0	MIR833	0	1	MIR840	1	0
MIR394	2	0	MIR856	0	1	MIR842	1	0
MIR396	2	0	MIR857	0	1	MIR844	1	0
MIR858	2	0	MIR868	0	1	MIR845_1	1	0
MIR162_1	1	0	MIR869	0	1	MIR846	1	0
MIR403	1	0	MIR774	2	0	MIR847	1	0
MIR408	1	0	MIR161	1	0	MIR848	1	0
MIR414	1	0	MIR163	1	0	MIR851	1	0
MIR482	1	0	MIR173	1	0	MIR852	1	0
MIR827_3	1	0	MIR1888	1	0	MIR853	1	0
MIR828	1	0	MIR2934	1	0	MIR859	1	0
			MIR3434	1	0	MIR860	1	0
			MIR3440	1	0	MIR861	1	0
Singletons	51	45	MIR391	1	0	MIR862	1	0

### Nonconserved *MIR*s showed higher intraspecies copy number variation than conserved *MIR*s

**Fig. 4:**
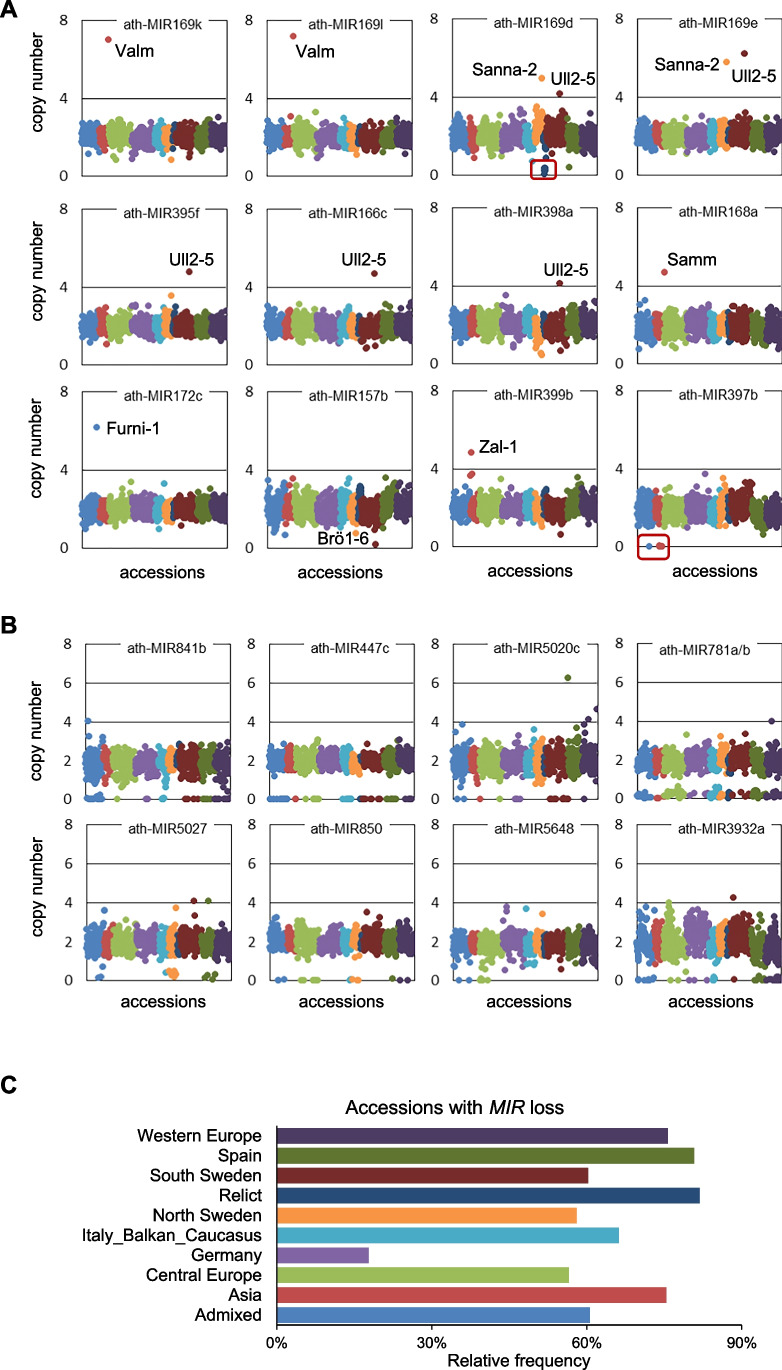
Copy number variations of conserved and nonconserved *MIR*s. **A** Duplications and deletions of conserved *MIR*s are rare or private variants. **B** Nonconserved *MIR*s in CNV regions are frequently deleted in a large number of accessions. Plots in A and B present copy number estimations for selected *MIR*s in 1060 accessions calculated based on short-read sequencing data coverage, as described in the main text. For the purpose of the genotype assignments, values lower than 0.3 were assumed to indicate copy losses, while the values equal to or higher than 4.0 were treated as copy gains. Accessions with gains or losses of conserved *MIR*s are indicated. **C** Rates of accessions with copy losses of nonconserved *MIR*s in genetic groups. Genetic groups were previously assigned to each accession within 1001 Genomes Project (1001 Genomes Consortium 2016) and are marked by different colours, identical for all panels. The source data for the plots, along with the accession names, IDs, and group assignments, are listed in the (Additional file 1: Supplementary Table S4)

A significant fraction, 76%, of the analysed conserved *MIR*s was nonvariable. Regarding the conserved *MIR* families, 15/29 of them were entirely nonvariable, while the remaining ones had from one to three individual variable *MIR*s (Table 1). Even for these *MIR*s, however, the changes affected only one or few accessions from the worldwide population (Fig. 4A). For example, *ath-MIR169k* and *ath-MIR169l* were the only variable *MIR*s among the seven members representing MIR169-1 family. They both showed copy gains in the same Russian accession Valm, most likely as a result of a private duplication, encompassing these two neighbouring *MIR*s. Similarly, two neighbouring members of MIR-169-2 family, namely, *ath-MIR169d* and *ath-MIR169e*, showed copy gain in two Swedish accessions—Sanna-2 and Ull2-5. Additionally, in Ull2-5, we detected copy gains for a total of five distinct conserved *MIR*s. It was distinguishable, since in any other accession, the number of conserved *MIRs* with duplications did not exceed 3, and 95% of accessions displayed no changes in the conserved *MIR*s copy number at all. We note that Ull2-5 also showed unusually high gain-to-loss ratio in our previous genome-wide analyses of CNVs spanning protein-coding genes (Zmienko et al. 2020); therefore, it seems to be a general feature, not restricted to specific *MIR*s. Further studies of Ull2-5, e.g. including long read-based genome sequencing, are required to understand the causes and possible functional implications of these duplications.

Remarkably, only four conserved *MIR*s were lost in some accessions, and all these deletions constituted private or rare variations. We only observed loss of *ath-MIR169d* in three relict accessions originating from Spain (Ip-Con-0, IP-Her12, and IP-Lso-0), loss of *ath-MIR157b* in one accession (Brö1-6), loss of *ath-MIR157d* in one accession (Stenk-4), and loss of *ath-MIR397b* in five accessions (Basta-2, Basta-3, Noveg-2, Noveg-3, IP-Hoy-0), mainly from Asia. Overall, we concluded that the conserved *MIRs* showed little intraspecies copy number variation. On the contrary, among *MIRs* belonging to the nonconserved families and singletons (to which we further collectively referred as the nonconserved *MIRs*), 45% displayed copy number changes. This result was consistent with the average overlap of the nonconserved *MIRs* by CNV regions. As discussed above, nonconserved *MIRs* overlapping protein-coding genes had substantially lower rates of copy number variation, compared to intergenic nonconserved *MIR*s. Regarding the individual variation patterns, some nonconserved *MIR*s displayed changes (mainly copy gains) restricted to only one or few accessions from a specific genetic group (Additional file 2: Supplementary Fig. S1). However, many *MIR*s showed a substantial diversity, manifested not only by the duplications but also by frequent deletions (Fig. 4B). There was a bias towards detecting more deletions in these accessions, which were distantly related to the reference genome. More specifically, we detected deletions of at least one nonconserved *MIR* in only 18% of accessions from the Germany group (to which Col-0 belongs), while for the remaining genetic groups, the number of accessions with deletions ranged from 57% for Central Europe to 82% for relicts (Fig. 4C). Regardless of the latter, for many nonconserved *MIR*s, the deletions affected accessions from various genetic groups, which suggested dispensability or functional redundancy of the respective miRNAs.

To evaluate the accuracy of our predictions regarding *MIR* copy number variation, we also performed multiplex ligation-dependent probe amplification (MLPA) assays, with the fully synthetic oligonucleotide probes (Marcinkowska-Swojak et al. 2013; Samelak-Czajka et al. 2017). We analysed 16 nonconserved *MIR*s, each in a set of 43–47 accessions (Additional file 1: Supplementary Table S5). For *ath-MIR4227*, the assay did not generate any signal in most accessions. Since MLPA requires a large number of samples for accurate group separation, this gene was excluded from the analysis. Additionally, for *ath-MIR773a*, we could not separate distinct groups, due to the noisy signal data. For *ath-MIR406* and *ath-MIR826a/b* as well as for *ath-MIR5022* and *ath-MIR3440b*, no copy changes were expected in the accessions assayed by MLPA. For *ath-MIR447c*, *ath-MIR833a*, *ath-MIR850*, *ath-MIR5027*, *ath-MIR5641*, *ath-MIR5648*, *ath-MIR5661*, and *ath-MIR8174*, varying number of accessions with copy losses were expected. Additionally, for *ath-MIR8174* and *ath-MIR5027*, single accessions with copy gains were expected. MLPA analysis confirmed these predictions in most cases (Additional file 2: Supplementary Fig. S2), with the following exceptions. We did not confirm copy gains for *ath-MIR8174* and *ath-MIR5027* by MLPA, while for *ath-MIR5641*, *ath-MIR5661*, *ath-MIR8174*, *ath-MIR850*, and *ath-MIR3440b*, we also observed the lack of MLPA signals in a small number of accessions, which were not identified in the bioinformatic predictions. In most cases, manual inspection of mapping reads in these regions in IGV browser provided explanation for these discrepancies (Additional file 3: Supplementary data). We also used MLPA to assess the variability of two genes that were filtered out from the bioinformatic analyses: *ath-MIR4239* and *ath*-*MIR8173*, the first one being located in the hotspot of rearrangement and the other one overlapping TE. For these two *MIR*s, the MLPA assays revealed a high ratio of accessions with gene deletions, indicating substantial copy number variation.

### *MIR*s involved in developmental control of transposon reactivation are mostly nonvariable

TE overlap constituted an important factor contributing to the *MIR* variation. Out of the 25 TE-associated *MIR*s which we were able to genotype, 20 were variable, often to a high level (Additional file 2: Supplementary Fig. S1). This was not surprising, however, since TEs themselves present substantial intraspecies variation (Quadrana et al. 2016) and could drive the variation of the *MIR*s which were derived from them. We next wanted to evaluate variability of *MIR*s that directly target transposons. Our bioinformatic analyses predicted that various TEs are targeted by 90 unique *MIR*s, including 40 TE-derived. Also in a previous study, about 50 *MIR*s/*MIR* families were identified, which together targeted more than 1200 transposons (Creasey et al. 2014). Similarly to our predictions, this included both TE-derived *MIR*s and other *MIR*s, some known to be developmentally regulated. It was demonstrated by Creasey and colleagues that these miRNAs specifically targeted transposon transcripts, but only when these transposons were reactivated during reprogramming of the germ line. In that case, miRNAs triggered epigenetically activated 21-nt siRNA biogenesis from transposons, dependent on RDR6, and inhibited 24-nt siRNA biogenesis processed by RDR2, which prevented long-term heterochromatic silencing of these transposons. We compared our CNV genotyping data with the list of transposon-targeting miRNAs identified by Creasey and colleagues. To our surprise, we found that the vast majority of them were nonvariable, regardless of their conservation level (Table 2). Thus, *MIR* role in controlling TE reactivation, which is a part of the plant developmental programme, may constitute an important selecting factor acting against accumulating copy number changes by these *MIR*s.

**Table 2:** List of *MIR*s involved in epigenetic silencing of reactivated transposons, taken from Creasey et al. (2014), divided by their conservativeness and variability

Conserved variable	Conserved nonvariable	Nonconserved variable	Nonconserved nonvariable
*ath-MIR167*^*a*^ *ath-MIR169*^*a*^ *ath-MIR2111*^*a*^ *ath-MIR390*^*a*^ *ath-MIR399*^*a*^	*ath-MIR156* *ath-MIR157a* *ath-MIR159* *ath-MIR162* *ath-MIR167*^*a*^ *ath-MIR169*^*a*^ *ath-MIR172b* *ath-MIR2111*^*a*^ *ath-MIR319* *ath-MIR390*^*a*^ *ath-MIR395* *ath-MIR396* *ath-MIR399*^*a*^ *ath-MIR858*	*ath-MIR418* *ath-MIR447*^*a*^ *ath-MIR5020* *ath-MIR5029* *ath-MIR833* *ath-MIR855* *ath-MIR863* *ath-MIR869*	*ath-MIR415* *ath-MIR420* *ath-MIR447*^*a*^ *ath-MIR5015* *ath-MIR5022* *ath-MIR771* *ath-MIR774* *ath-MIR775* *ath-MIR823* *ath-MIR825* *ath-MIR834* *ath-MIR838* *ath-MIR843* *ath-MIR847* *ath-MIR859* *ath-MIR861* *ath-MIR862* *ath-MIR866*

^a^Whenever data provided by Crasey et al. did not specify the family member and this family had both variable and nonvariable members, we included it in both groups

### Global differences in miRNA production are related to *MIR* conservation level rather than variability

**Fig. 5:**
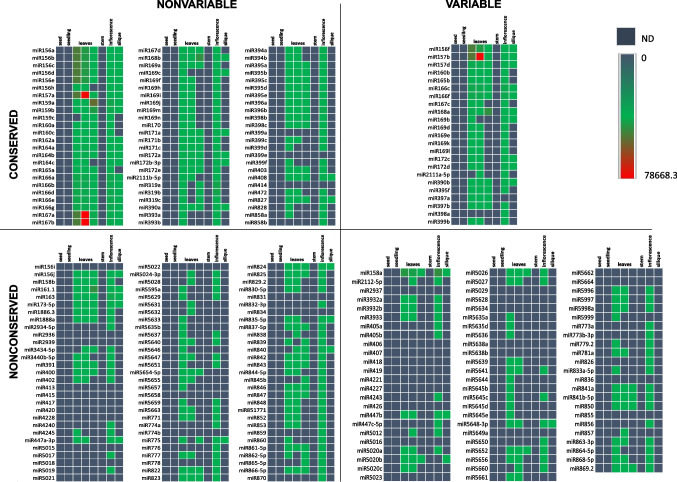
Variable and nonvariable *MIR*s expression in Col-0 accession. NGS sRNA-seq data for mature miRNA accumulation, presented as RPM (reads per million) counts normalized to all miRNAs identified in the sample, were downloaded from mirEX^2^ database (Zielezinski et al. 2015). *MIR*s are grouped by their conservation and copy number variability

To assess whether copy number diversity affects *MIR* expression, we evaluated mature miRNA expression profiles in Col-0, using processed data downloaded from mirEX^2^ database (Zielezinski et al. 2015). We analysed expression data from seeds, seedlings, rosette leaves, stems, inflorescence, and siliques. Globally, miRNA production was highest in the leaves and inflorescence, both in terms of miRNA levels and the number of detectable miRNAs (Fig. 5). In these organs, most conserved miRNAs could be detected, and their expression levels were higher than that of nonconserved *MIR*s, regardless of their variability. Among the nonconserved *MIRs*, the number of mature miRNA that could be detected in leaves, inflorescence, and siliques was always higher than the number of detectable nonconserved variable miRNAs (Additional file 2: Supplementary Fig. S3). This could mean that numerous nonconserved variable *MIR*s have very specific expression profiles or that their loci are mostly silent, e.g. due to genomic DNA methylation.

Next we checked whether we could directly detect the impact of *MIR* variations on mRNA accumulation. We used the publicly available gene expression data from rosette leaves for 22,611 genes in 601 accessions in common with our study (Kawakatsu et al. 2016). We calculated the correlations between the copy numbers of 103 variable *MIR*s and the expression of protein-coding genes across the accessions (Additional file 1: Supplementary Table S6). However, in most cases, we were not able to observe any association. Only for *ath-MIR447c* and *ath-MIR864*, we observed some negative correlation of the genotyping data with the expression of one and eight protein-coding genes, respectively (Pearson correlation coefficient *r* ranged from − 0.30 to − 0.36, which passed the significance threshold set at − 0.3). However, these mRNAs were not among the predicted targets of *ath-MIR447c* or *ath-MIR864*. Surprisingly, we also observed some positive correlations (Pearson correlation coefficient r ranged from 0.30 to 0.78) with the expression of protein-coding genes for seven *MIR*s (e.g. *ath-MIR5661* and a hypothetical protein-coding gene *AT4G19080*; Pearson correlation coefficient *r* = 0.52). We were able to partially explain these results by the similarities of copy number variation patterns of these gene pairs, which highlighted technical difficulty in distinguishing between the effect of these elements proximity in the genome and their potential functional relation, with the limited data.

## Discussion

*MIR*s expression is regulated on the transcriptional and posttranscriptional levels by multiple standard mechanisms, such as polyadenylation or splicing, to ensure the required abundance of particular miRNAs (Wang et al. 2019; Li and Yu 2021; Bhogireddy et al. 2021; Zhang et al. 2022). Frequently, miRNAs participate in the regulation of important developmental and physiological processes, e.g. tissue differentiation, organ formation, biotic and abiotic stress responses, signal transmission, metabolism, or transition from vegetative to generative phase (Gupta et al. 2017; Song et al. 2019; Ó’Maoiléidigh et al. 2021; Yang et al. 2021; Betti et al. 2021; Brosnan and Mitter 2021; Öztürk Gökçe et al. 2021; Pradhan et al. 2021; Dong et al. 2022; Jeena et al. 2022). Thus, maintaining intracellular miRNA homeostasis is crucial for the organism, and both over- and underproduction of miRNAs may influence various biological processes. This influence may be noticeable especially in the case of the conserved miRNAs, the disregulation of which might have a strong effect on plant fitness. For example, *MIR319a* is a member of the conserved MIR159 family and encodes miRNA that targets *TCP4* transcription factor gene transcript. In Arabidopsis, *ath-MIR319a* loss-of-function allele triggered the defects in petal and stamen development (Nag et al. 2009), while ectopic expression of this *MIR* resulted in the uneven leaf shape and curvature (Palatnik et al. 2003). In other plants, such as rice, tomato, or creeping bentgrass, constitutive expression of *MIR319* also affected leaf morphology and led to increased plant tolerance to cold, drought, or salinity (Zhou et al. 2013; Yang et al. 2013; Shi et al. 2019). Likewise, increased expression of a conserved *ath*-*MIR408* gene conferred better cold, salinity, and oxidative stress tolerance in Arabidopsis, but enhanced its sensitivity to drought (Ma et al. 2015). Overexpression of Arabidopsis, rice, or tobacco *MIR408* also improved the vegetative growth and seed yield in the respective plant species (Pan et al. 2018). These examples indicate the importance of the conserved miRNA-mediated regulation in the posttranscriptional control of genes involved in plant growth and adaptation. Consistent with these findings and with the confirmed role of CNVs in altering gene structure, dosage, and transcriptional regulation (Żmieńko et al. 2014; Shao et al. 2019; Garg et al. 2022), we found that the conserved *MIR*s presented very low levels of intraspecies copy number diversity (24%), and none of them overlapped with any TE.

We found that protein-coding genes and TEs oppositely affected the polymorphism rate of nonconserved *MIR*s derived from them. While the majority of nonconserved *MIR*s which overlapped with the protein-coding genes were nonvariable, TE-derived *MIR*s presented exceptionally high levels of diversity. Although many of them were filtered out from our analysis, bioinformatic genotyping and the MLPA assays indicated that these genes also represented the variable fraction of the nonconserved *MIR*s. Our difficulties with the analysis of *MIR*s overlapping TEs and the hotspots of rearrangements were thus not surprising, considering the well-known shortcomings of short-read sequencing data when applied to the genomic regions of high structural complexity (Medvedev et al. 2009).

TEs are considered to be one of the main drivers of gene copy number variation (Chen et al. 2014; Chu et al. 2021). The role of TEs in the expansion of some *MIR*s in rice, bread, wheat, and cotton has been demonstrated previously (Li et al. 2011; Shen et al. 2020; Campo et al. 2021; Crescente et al. 2022). In agreement with these findings, our data suggested that the emergence and evolution of some recent *MIR* families in Arabidopsis were associated with the mechanisms triggering amplification of specific TEs (Naish et al., 2021). Accordingly, we showed that TEs belonging to DNA/MuDR family, which is the most abundant MITE family found in Arabidopsis—and MITEs are frequent sources of de novo *MIRs* (Guo et al. 2022)—are enriched among the *MIR*-overlapping TEs.

On the other hand, *MIR*s known to target transposons epigenetically reactivated during germ line reprogramming tend to be nonvariable (Table 2), regarding of their conservation level, which strongly indicates that copy number changes might interfere with the functionality of variable *MIR*s. While the effect of copy gains on gene expression might be mitigated by numerous genetic and epigenetic regulatory mechanisms (Wolffe and Matzke 1999; Lee and Chen 2001; Keller and Yi 2014; Ascencio et al. 2021), gene loss is an irreversible process, and its functional impact might be much greater. Consistent with the preservation of conserved *MIR*s across the plant kingdom and their frequent involvement in plant development, deletions of these *MIR*s were extremely rare. On the contrary, copy number changes, most prominently deletions, affected about 45% of the nonconserved *MIR*s. This might indicate that these *MIRs* are dispensable or perform redundant functions, which can be taken over by other members of *MIR* family. Indeed, *MIR*s representing nonconserved families with many members frequently presented copy number diversity (Table 1). High rate of intraspecies deletions of some nonconserved *MIR*s indicated that they have not been fixed in the Arabidopsis genome. Altogether, our results showed that the mechanisms inducing copy number changes are engaged in the ongoing dynamic evolution of the nonconserved *MIR*s.

Evaluation of mature miRNA accumulation in the reference accession Col-0 revealed that the global pattern of *MIR* expression is highly dependent on their conservation level and to a lesser extent on their variability. This finding is in agreement with the known involvement of conserved *MIRs* in basic processes, e.g. controlling plant development. In the genome-scale comparisons, we were not able to find direct links between the copy number of any *MIR* with the mRNA variation in Arabidopsis leaves. This does not rule out the possibility that *MIR* copy number variation affects leaf transcriptome, since this effect may be more subtle. While it can be expected that the copy number alteration of a *MIR* gene would affect the expression of the target gene in a dosage-dependent manner, miRNA-mediated regulation is only one of the many mechanisms which together contribute to the observed gene expression level. Therefore, dedicated functional studies are needed to establish whether—and to what extent—*MIR* structural variation might affect the amounts of their target transcripts. Moreover, combining *MIR* variation data with the intraspecies gene expression datasets generated for various tissues or conditions may be needed, depending on the expression profiles of individual *MIR*s of interest. For many variable MIRs, there is currently not enough information regarding their expression and validated targets to perform such studies.

Finally, our CNV analysis was based on mapping short reads to the common reference genome (The Arabidopsis Genome Initiative 2000). A known bias of such an approach is that more deletions will be detected in the accessions which are distantly related to the used reference, due to their higher sequence divergence. Indeed, we observed such an effect in our analysis. By analogy, any *MIR*s, which were present in the nonreference accessions only, were not studied by us. So far, most Arabidopsis *MIR*s were detected with the reference-based approaches (Wang et al. 2004; Zhu et al. 2020; Mehdi et al. 2021); therefore, it can be anticipated that many *MIR*s are still missing from the present miRBase catalogue. With the advent of third-generation sequencing methods and the substantial increase in the number of de novo assembled genomes, representing different individuals of the same species, our knowledge about the genome diversity in plants and animals will undoubtedly increase in the near future. These new approaches will also allow us to combine the assessment of the *MIR* numbers and genomic localizations of individual copies as well as their exact nucleotide sequences and epigenetic statuses, in order to get more detailed information about their birth and evolution as well as the types and the frequency of *MIR* polymorphisms, or their putative roles in plant growth and adaptation.

## Materials and methods

### Data sources and genomic datasets

A list of Arabidopsis *MIR*s with coordinates, mature miRNA sequences, and family information was downloaded from the miRBase database (Release 22.1: October 2018) (Kozomara et al. 2019). For each *MIR* family, the conservation was assessed based on the information about the occurrence of its members in different taxa, which was also retrieved from the miRbase database (Additional file 1: Supplementary Table S1). The coordinates of protein-coding genes and TEs were according to Araport11 version of the TAIR10 genome annotation (Cheng et al. 2017). Centromere coordinates were according to Clark et al. (2007). The list of Arabidopsis CNV variants with coordinates was from Zmienko et al. (2020). The associated information about protein-coding genes copy number estimations was downloaded from http://athcnv.ibch.poznan.pl/. Whole-genome sequencing data for 1060 accessions were downloaded from the National Center for Biotechnology Information Sequence Read Archive repository (PRJNA273563; https://www.ncbi.nlm.nih.gov/bioproject/PRJNA273563). RNA-seq data (normalized counts) for 728 accessions were downloaded from the Gene Expression Omnibus repository (PRJNA319904; https://www.ncbi.nlm.nih.gov/bioproject/PRJNA319904). Illumina sequencing sRNA-seq data for mature miRNA accumulation, presented as RPM (reads per million) counts normalized to all miRNAs identified in the sample, were downloaded from mirEX^2^ database (Zielezinski et al. 2015). Col-CEN de novo assembly of the Arabidopsis genome (assembly ASM2311539v1; GenBank accession GCA_023115395.1) was directly applied for online blastn searches using NCBI internal search and retrieval system.

### *MIR*s copy number genotyping

Short-read sequencing data from Arabidopsis 1001 Genomes Project (1001 Genomes Consortium 2016) were downloaded from National Center for Biotechnology Information Sequence Read Archive repository (PRJNA273563) and processed and mapped to the reference genome as described in Zmienko et al. (2020). For each *MIR*, the genomic coordinates of the region used in genotyping were defined by extending the coordinates of the stem-loop precursor by 300 nt from each site. *MIR*s overlapping each other were genotyped as one region. This included seven *MIR* pairs: *ath-MIR5998a* and *ath-MIR5998b*, *ath-MIR826a* and *ath-MIR826b*, *ath-MIR2934* and *ath-MIR782*, *ath-MIR5595a* and *ath-MIR5595b*, *ath-MIR833a* and *ath-MIR833b*, *ath-MIR5649a* and *ath-MIR5649b*, and *ath-MIR781a* and *ath-MIR781b*. Genotyping of 1060 accessions was performed with Genome STRiP SVGenotyper (Handsaker et al. 2015). Genome STRiP is a suite of tools for discovery and genotyping of structural variation using whole-genome sequencing data in a population-based approach; i.e. they are designed to find shared variation using data from multiple individuals. For *MIR* copy number estimations, we followed the procedure described in Zmienko et al. (2020), using the extended genomic coordinates of *MIR* genes, described above. The nonunique segments in the reference genome, found by creating subsequence strings with 40-bp sliding windows and a 1-bp step and aligning them with the reference genome, were masked prior to genotyping, to increase the accuracy of genotyping highly similar paralogs (Handsaker et al. 2015). The analysis failed for 7 *MIR*s, and they were removed from the analysis. For the remaining *MIR*s, unrounded copy number estimates in all accessions were processed further by applying custom filters for marking and removing *MIR*s with untypical copy number distributions. More specifically, we filtered out all *MIR*s, for which at least one of the following criteria was true: (i) unrounded copy number in Col-0 was lower than 1.0 or higher than 3.0; (ii) mean unrounded copy number in the population of 1060 accessions was lower than 1.0 or higher than 3.0; and (iii) the (upper quartile minus lower quartile) range value for copy number distribution was higher than 1. The filtering procedure resulted in the removal of additional 40 *MIR*s, leaving 271 *MIR*s with the genotyping data. For the purpose of the genotype assignments, values lower than 0.3 were assumed to indicate copy losses, while the values equal to or higher than 4.0 were treated as copy gains. The genotyping results are presented in Additional file 1: Supplementary Table S4.

### Plant materials and DNA extraction

Arabidopsis seeds were obtained from the Nottingham Arabidopsis Stock Centre. The seeds were surface-sterilized and vernalized for 3 days. Subsequently, the seeds were planted on Jiffy pellets (BETATECH) and grown in a growth chamber under long day conditions (16-h light; 8-h dark; 22 °C/18 °C, 70% humidity) with nourishment from 0.5 Murashige and Skoog medium (Serva). The leaves of 3-week-old plants were cut, immediately frozen in liquid nitrogen, and stored at − 80 °C. Genomic DNA was extracted using the DNeasy Plant Mini Kit (Qiagen), with RNase A treatment step. The sample concentration and quality were assessed on a Nanodrop 2000 spectrophotometer (Thermo Scientific). The list of accessions used for the experimental verification is provided in Additional file 1: Supplementary Table S5.

### Multiplex ligation-dependent probe amplification (MLPA) assays

The MLPA assays were performed exactly as described previously, using 5 ng DNA templates (Samelak-Czajka et al. 2017). The left and right target-specific sequences (TSSs) of the MLPA probes targeted the genomic regions within the stem-loop sequence of the respective *MIR*s (Additional file 1: Supplementary Table S7). The only exception was a*th-MIR8174*, for which the MLPA probe targeted the 3’ boundary of the predicted pri-miRNA region. To ensure optimal performance of the MLPA assays, regions rich in single-nucleotide variants were avoided during the probe design, as verified by examining the vcf files for 1135 accessions, obtained from the 1001 Genomes Project website (1001 Genomes Consortium 2016). Probe specificity was verified with blastn against the Arabidopsis genome (TAIR10). The MLPA assays were performed with the SALSA MLPA reagent kit (MRC-Holland). A uniform set of 48 accessions was selected for the analysis, including accessions with the predicted duplications/deletions in the assayed *MIR* loci, as indicated by the bioinformatic genotyping results. The MLPA amplification products were separated by capillary electrophoresis on an ABI Prism 3130XL Genetic Analyzer (Applied Biosystems). The results were analysed with the GeneMarker v.3.0.1 software (SoftGenetics). The signal intensity for each probe was normalized to the average of the three control probes. The normalized signal intensity threshold values for detecting deletions and duplications were set at 0.5 and 2.0, respectively. The results are presented in Additional file 1: Supplementary Table S5.

### Gene expression correlation analysis

Preprocessed and normalized RNA-seq data (Kawakatsu et al. 2016) for 728 accessions were downloaded from the Gene Expression Omnibus repository (https://www.ncbi.nlm.nih.gov/bioproject/PRJNA319904). Normalized gene expression counts for all protein-coding genes were used for calculating correlation with the *MIR* copy number estimates, in 601 accessions, common form both datasets. Pearson correlation coefficient (*r*) higher than 0.3 or lower than − 0.3 was used as a threshold for detecting significantly high positive and negative correlation, respectively. The results are listed in Additional file 1: Supplementary Table S6. Targets for all miRNAs were predicted with small RNA target prediction tool (https://wasabi.ddpsc.org/~apps/tp/) (Jones-Rhoades and Bartel 2004; Nakano et al. 2020), using mature miRNA sequences from the miRBase (release 22.1) as queries and Arabidopsis TAIR10 cDNA set as the target database, with the default parameters. This procedure resulted in predicted targets for 311 *MIR*s. The results are listed in Additional file 1: Supplementary Table S2.

## Supplementary Information

Additional file 1 – contains Supplementary Tables S1-S7; .xlsx file (XLSX 37349 kb)

Additional file 2 – contains Supplementary Figures S1-S3; .pdf file (PDF 294 kb)

Additional file 3 – contains Supplementary Data; .pdf file (PDF 157 kb)

## Data Availability

Data generated or analysed during this study are included in this published article and its supplementary information files. Public sources of previously published WGS, RNA-Seq datasets, and CNVs are detailed in the Materials and Methods section.
