# Supplementary material for: Differences in the intraspecies copy number variation of Arabidopsis thaliana conserved and nonconserved miRNA genes
Source: Funct Integr Genomics. 2023 Apr 10;23(2):120. doi: 10.1007/s10142-023-01043-x (PMC10085913; doi:10.1007/s10142-023-01043-x)
Supplement: Supplementary file 2 — – contains Supplementary Figures S1-S3; .pdf file (PDF 294 kb) [file 10142_2023_1043_MOESM2_ESM.pdf]

A

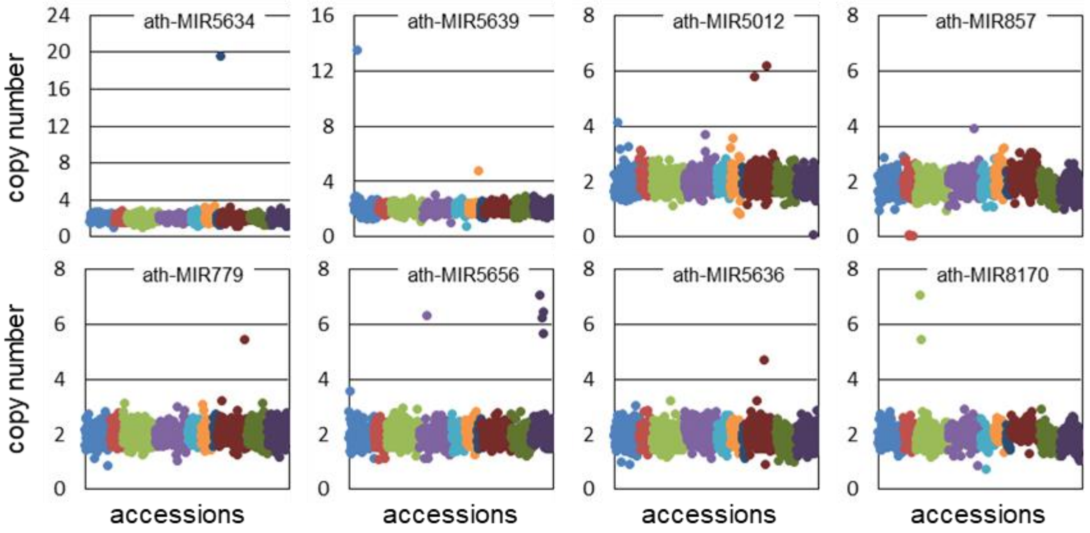

B

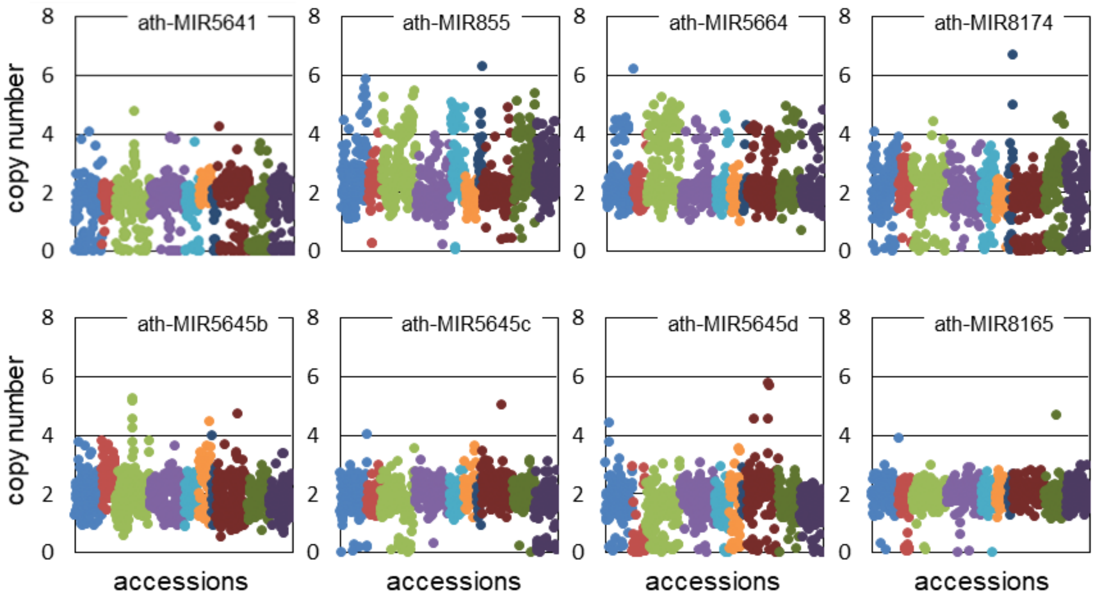

Supplementary Fig. S1 Exemplar plots nonconserved *MIR*s. A) Nonconserved *MIR*s with rare copy number changes. B) Copy number-variable TE-associated *MIR*s. Source data for plots are in Additional file 1: Supplementary Table S4.

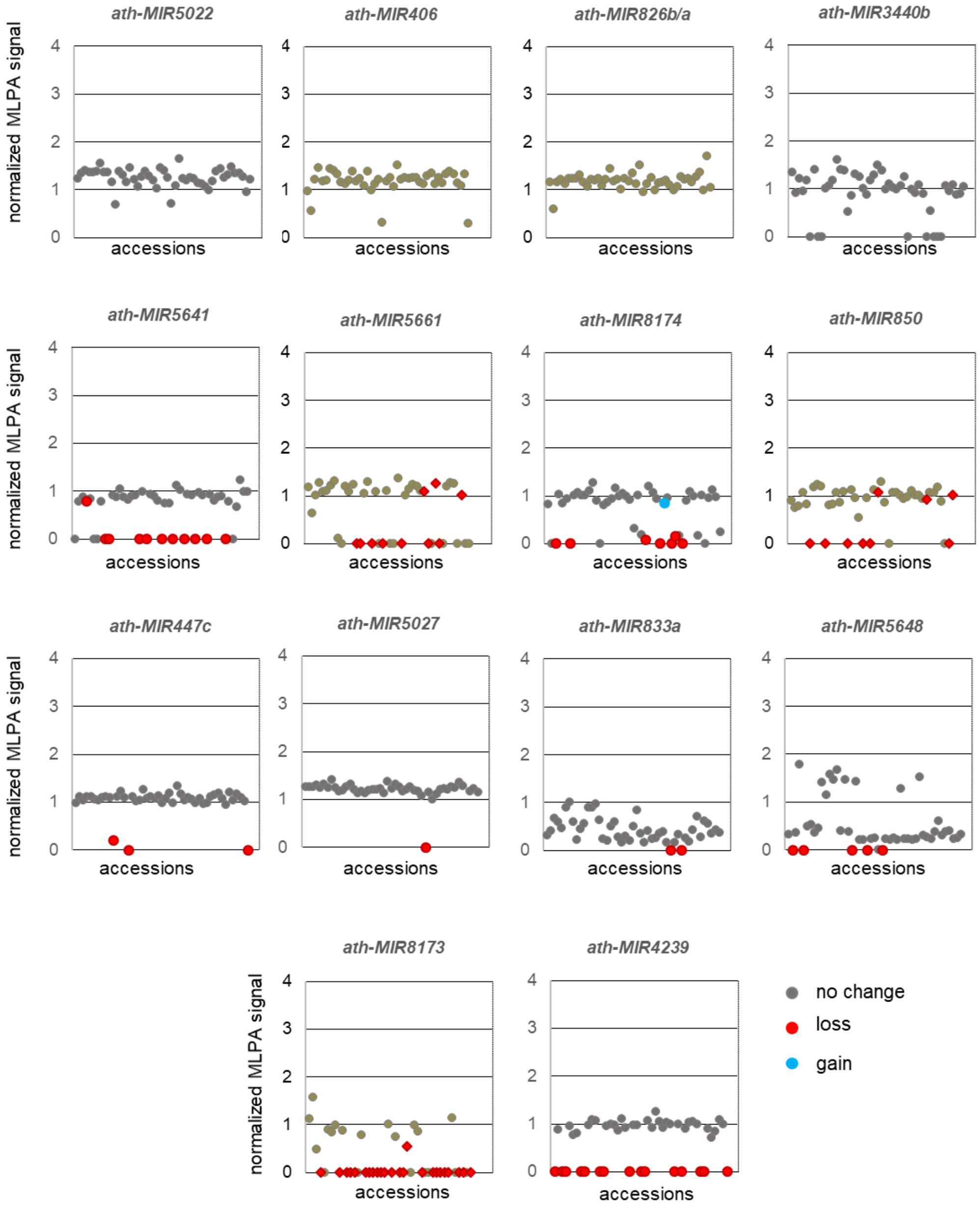

Supplementary Fig S2 MLPA results for individual *MIRs*. Each plot presents normalized MLPA signal for 43-47 accessions, listed in Additional file 1: Supplementary Table S5. Colors denote the genotypes assigned by the bioinformatic analysis of read coverage data.

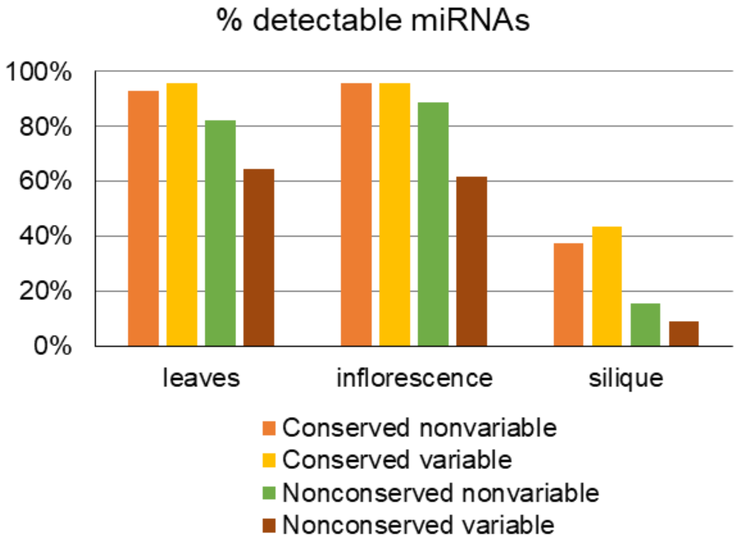

Supplementary Fig S3 Percentage of detectable mature miRNAs in Col-0 accession for *MIR*s differing in the conservation level and variability. Source data were downloaded from the mirEX^2 database (Zielezinski et al. 2015). For plotting, NGS sRNA-seq data (normalized RPMs) were used. miRNAs with non-zero levels were treated as detectable. Accumulation levels for individual miRNAs are presented in Fig 5 in the main text.
