# Supplementary material for: Differences in the intraspecies copy number variation of Arabidopsis thaliana conserved and nonconserved miRNA genes
Source: Funct Integr Genomics. 2023 Apr 10;23(2):120. doi: 10.1007/s10142-023-01043-x (PMC10085913; doi:10.1007/s10142-023-01043-x)
Supplement: Supplementary file 3 — – contains Supplementary Data; .pdf file (PDF 157 kb) [file 10142_2023_1043_MOESM3_ESM.pdf]

Supplementary data

Evaluation of discrepancies between MLPA assays and Genome STRiP genotyping results

Short genomic reads mapped to the reference genome were retrieved for the *MIR*s and accessions. Screenshots present genomic regions used for Genome STRiP genotyping (*MIR* with 300-bp flanking regions). *MIR* loci are in blue. Regions targeted by MLPA probes are in red. Accessions' names are shown in the left panel, along with the copy number estimations obtained with the Genome STRiP tool. For all presented cases, MLPA assays reported no signal.

ath-MIR5641

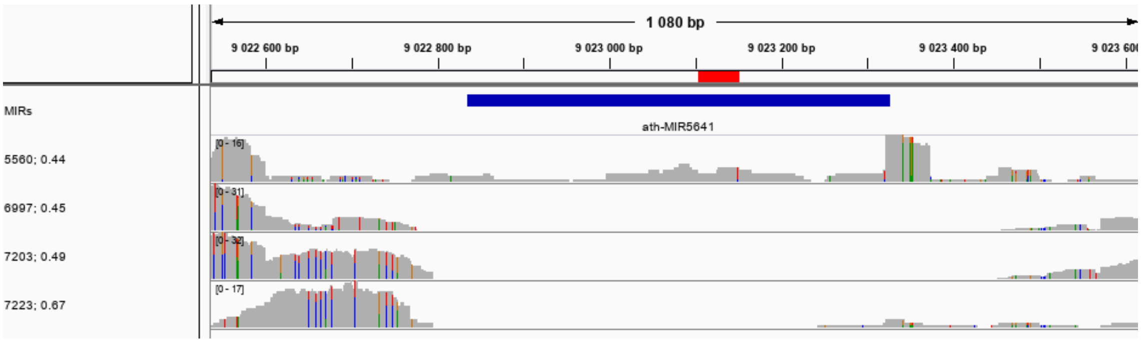

Comment: for these accessions, Genome STRiP estimations were much lower than for the rest of the population, however, they did not fulfil the deletion threshold value, which was globally set at 0.3.

ath-MIR5661

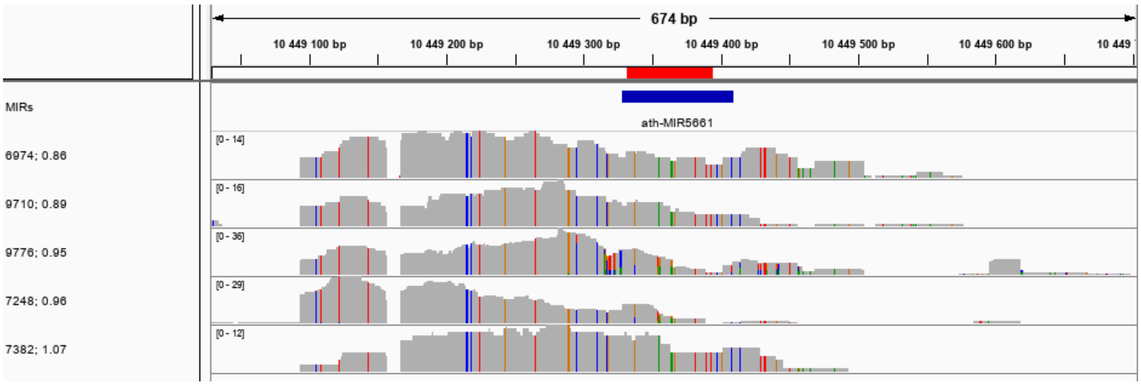

Comment: for these accessions, Genome STRiP estimations were lower than for the rest of the population, however, they did not fulfil the deletion threshold value, which was globally set at 0.3. Numerous SNPs present in the mapped reads indicate structural divergence (which might have affected probe hybridization) or mis-mapping of reads derived from a homologous genomic region, which is a known limitation of the reference genome mapping-based analysis.

ath-MIR3440b

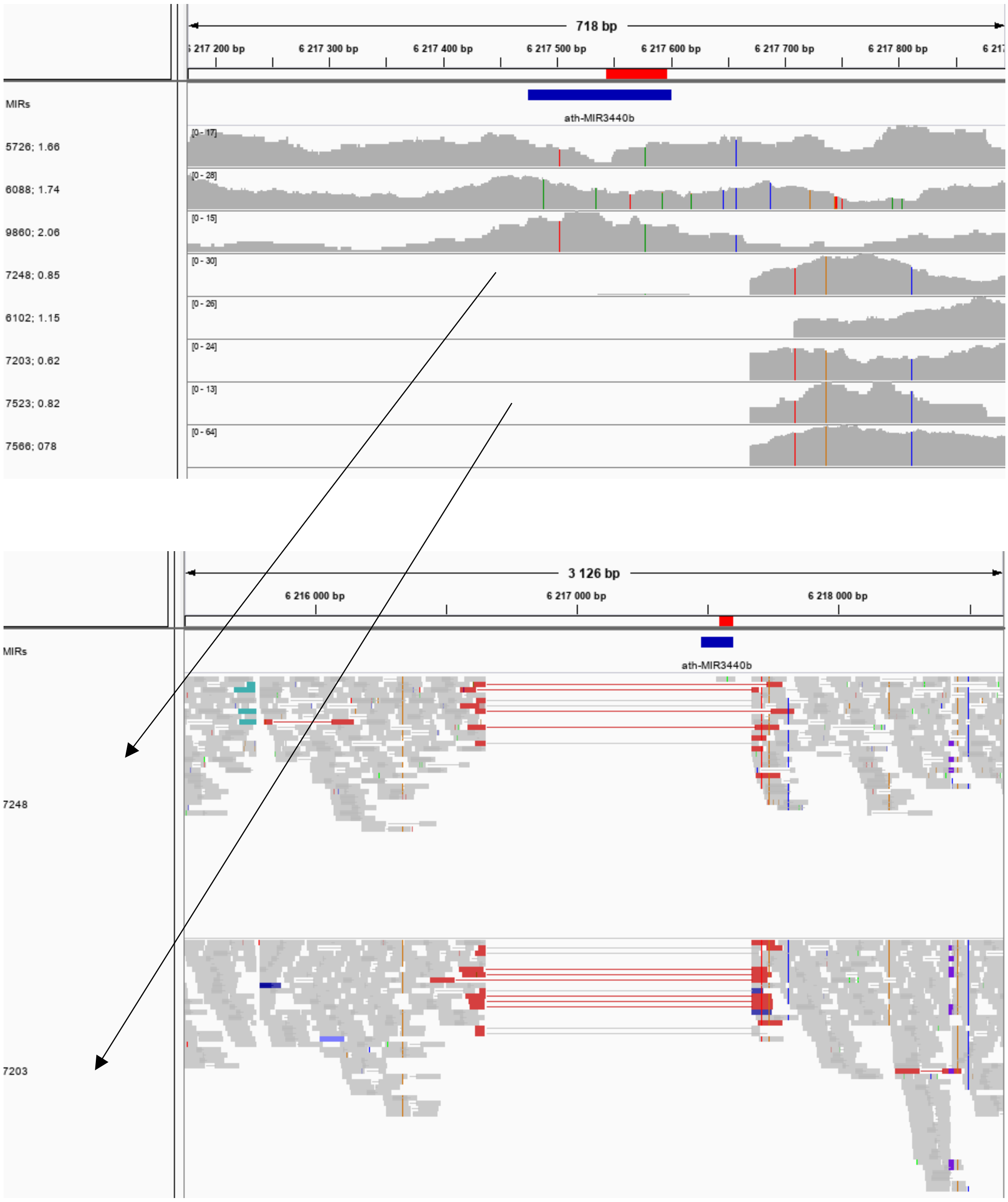

Comment: for three accessions, there are SNPs present around the MLPA half-probes' ligation region, which might have affected performance of the MLPA assay. For the remaining five accessions, mapping data indicate that a large part of the genotyped region, including *MIR* locus was actually missing, however reads mapping to the surrounding sequence contributed to erroneous estimation of this *MIR* as present.

ath-MIR8174

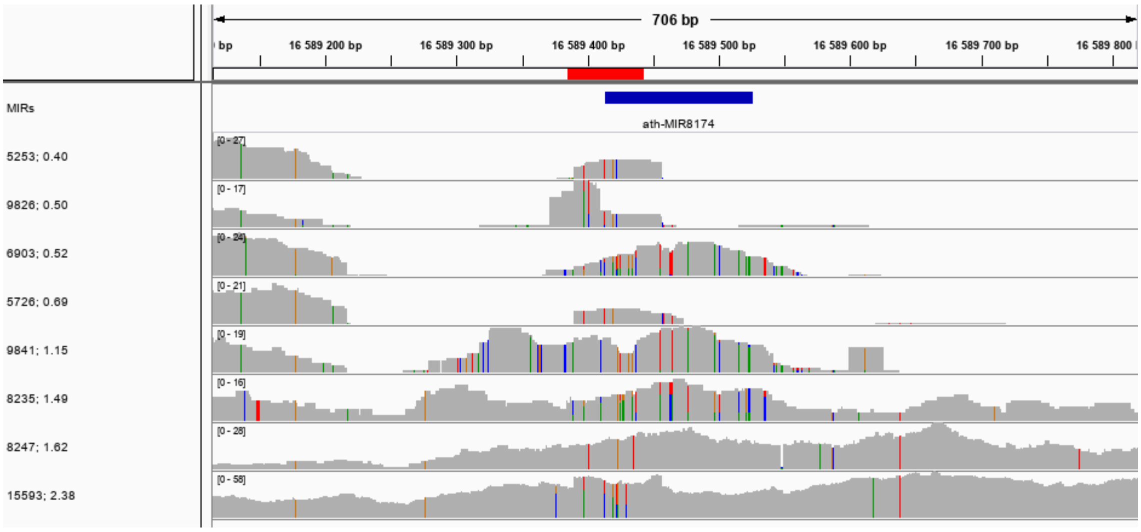

Comment: for this *MIR*, MLPA probe was designed at the edge of *MIR* locus, for specificity reasons. In the accessions shown above, numerous SNPs are present in mapped reads. This indicate structural divergence of this locus (which might have affected probe hybridization) or mis-mapping of reads from a homologous genomic region, which is a limitation of the reference genome mapping-based analysis. For five accessions, Genome STRiP estimations were lower than for the rest of the population, however, they did not fulfil the deletion threshold value, which was globally set at 0.3.

ath-MIR850

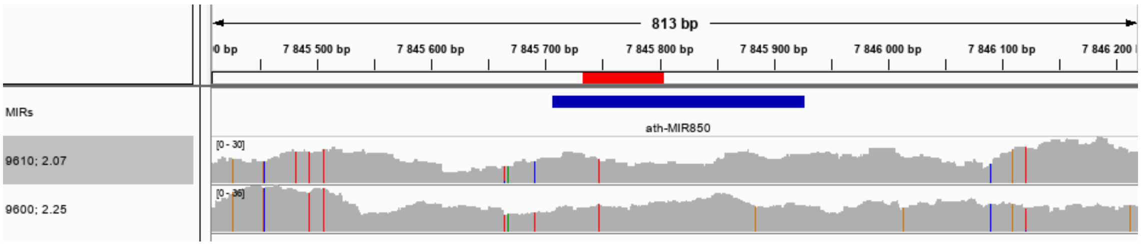

Comment: in these two accessions there are some SNPs in the region targeted by MLPA probes, however they are not supposed to seriously affect probe hybridization. MLPA probe covers only a small part of *MIR* locus and bioinformatic prediction utilized region extended beyond this locus, therefore resolving these discrepancies requires additional information, not dependent on the reference genome sequence.
